# Enhanced Production of Ligninolytic Enzymes by a Mushroom *Stereum ostrea*

**DOI:** 10.1155/2014/815495

**Published:** 2014-12-30

**Authors:** K. Y. Usha, K. Praveen, B. Rajasekhar Reddy

**Affiliations:** ^1^Department of Microbiology, Sri Krishnadevaraya University, Anantapur, Andhra Pradesh 515003, India; ^2^Department of Medical Microbiology, Hawassa University, P.O. Box 5, Hawassa, Ethiopia

## Abstract

The white rot fungi *Stereum ostrea* displayed a wide diversity in their response to supplemented inducers, surfactants, and copper sulphate in solid state fermentation. Among the inducers tested, 0.02% veratryl alcohol increased the ligninolytic enzyme production to a significant extent. The addition of copper sulphate at 300 *μ*M concentration has a positive effect on laccase production increasing its activity by 2 times compared to control. Among the surfactants, Tween 20, Tween 80, and Triton X 100, tested in the studies, Tween 80 stimulated the production of ligninolytic enzymes. Biosorption of dyes was carried out by using two lignocellulosic wastes, rice bran and wheat bran, in 50 ppm of remazol brilliant blue and remazol brilliant violet 5R dyes. These dye adsorbed lignocelluloses were then utilized for the production of ligninolytic enzymes in solid state mode. The two dye adsorbed lignocelluloses enhanced the production of laccase and manganese peroxidase but not lignin peroxidase.

## 1. Introduction

Most textile industries produced a large amount of wastewater with the excessive colour. Most of textile waste water contained approximately 20–200 mg/L of dye that could degrade the water quality [1]. White rot fungi are well known for their outstanding decolourization ability of synthetic dyes mediated by their oxidative ligninolytic complex [2], lignin peroxidase (LiP), manganese dependent peroxidase (MnP), and a family of multicopper oxidases, namely, laccases (Lcc).

Ligninolytic enzymes have a potential in several industrial and biotechnological processes [3, 4] including delignification of lignocellulosic biomass for fuel (ethanol) production; food, brewery, and wine; animal feed; denim stone washing; laundry detergents; paper and pulp industries; and bioremediation of chemical pollutants [5, 6]. Due to the potential applications of these enzymes, research in this area is oriented towards the search for efficient production systems. Reducing the cost of enzyme production by using cheaper raw materials and optimizing the fermentation process for industrial purposes is the ultimate target of basic research [7, 8]. A good strategy for this purpose is the production of these enzymes by solid state fermentation (ssf) technique using agroindustrial wastes as a support substrate. Most of such wastes are rich in soluble carbohydrates and also contain inducers of laccase synthesis, ensuring an efficient production of these enzymes [9]. SSF processes have shown to be particularly suitable for the production of enzymes by filamentous fungi, since they reproduce the natural living conditions of such fungi due to which they may be more capable of producing certain enzymes with high productivity in comparison to submerged fermentation [10].

Because of the diverse applications of ligninolytic enzymes in industrial processes, there is a wide interest in the induction, enhancement, and stabilization of these enzymes. The production of ligninolytic enzymes can be stimulated by the presence of a wide variety of inducing substrates mainly aromatic or phenolic compounds related to lignin or lignin derivatives such as ferulic acid, 2,5-xylidine, and veratryl alcohol [11]. Copper as a micronutrient has a key role as a metal activator, induces both laccase transcription, and plays an important role in laccase production [12]. Surfactants can stimulate the growth of spores and increase the bioavailability of less soluble substrates for the fungus thereby increasing the production of enzymes [13]. Hence the effect of these three types of compounds on enzymes was studied to evaluate their importance in getting maximum yields of ligninolytic enzymes.

As stated earlier, these enzymes are capable of decolorizing a variety of synthetic dyes. Effective decolorization of dyes is achieved by the integration of two methods—biosorption and biodecolorization. Biosorption by lignocelluloses may be an alternative method for removing dyes from effluents. Lignocellulosic biomass has potential of biosorption of various textile dyes. These dye adsorbed lignocelluloses could be utilized for the production of ligninolytic enzymes in solid state mode. The combination of biosorption and ssf of dye adsorbed lignocelluloses creates an effective method for dye removal and enzyme production.

## 2. Materials and Methods

The fungus was kindly supplied by Professor M. A. Singaracharya, Department of Microbiology, Kakatiya University, Andhra Pradesh, India, and was isolated from wood logs. The isolate was maintained at 4°C on 2% Koroljova-Skorobogat'ko medium [14] because of good growth. The maintenance medium was prepared according to Koroljova-Skorobogat'ko et al., (1998) containing the following composition (g/L): 3.0 peptone, 10.0 glucose, 0.6 KH_2_PO_4_, 0.001 ZnSO_4_, 0.4 K_2_HPO_4_, 0.0005 FeSO_4_, 0.05 MnSO_4_, 0.5 MgSO_4_, and 20.0 agar (pH 5.5).

### 2.1. Influence of Different Compounds on Enzyme Production

Duplicate flasks containing 5 g of wheat bran moistened with Koroljova medium (70% w/v) were used as production medium to carry out the following experiments.

(i) Different inducers were screened to get higher enzyme production from the culture of* S. ostrea*. Inducers like guaiacol (0.02%), veratryl alcohol (0.02%), lignin (0.1%), lignosulfonic acid (0.1%), gallic acid (0.02%), and tannic acid (0.05%) were amended in the production medium and sterilized. (ii) To find out the suitable concentration of copper sulphate for the maximum production of laccase, different concentrations of CuSO_4,_ 30, 50, 100, 300, 500, and 1000 *μ*M, were added to the production medium and sterilized. (iii) To study the influence of surfactants, 1 mL of different surfactants like Tween 20, Tween 80, and Triton X-100 was added at 1% conc. to the sterilized medium at the time of inoculation.

All the above flasks were aseptically inoculated with 12 mycelial plugs (7 mm) of 7-day-old culture and incubated at 30°C for a period of 12 days. Enzymes were extracted by adding 25 mL of phosphate buffer (pH 7.0; 100 mM) to each culture flask and kept on a temperature controlled gyratory shaker (ORBITEK-Chennai, India) (180 rpm) at 30°C for 1 hour. The mixtures were filtered through a sterile cotton cloth and the filtrate obtained was centrifuged (REMI C-24 BL) at 10,000 rpm at 4°C for 20 min. The supernatant obtained was analyzed for enzyme activities and extracellular proteins.

### 2.2. Biosorption and Biodecolorization of Dyes

Remazol brilliant blue (RBB) and remazol brilliant violet 5R (RBV) purchased from Sigma were used in the present study. 5 g of wheat bran/rice bran (RB/WB) and 100 mL of each dye (50 ppm) were taken into separate flasks and incubated at 30°C and 150 rpm for 30 min. After incubation period the suspensions were centrifuged at 5000 rpm for 15 min and then the supernatant solutions were analyzed for adsorption of dyes by monitoring the absorbencies at their *λ*_max⁡_ and adsorption was expressed in terms of percentage compared with control. For solid state fermentation studies Erlenmeyer flasks containing 5 g of dye adsorbed lignocelluloses moistened with Koroljova broth (70%) were sterilized and inoculated with 15 mycelial plugs (7 mm) and incubated for 15 days at 30°C under static conditions. Extraction of enzymes was carried out as mentioned above.

### 2.3. Enzyme Assay

Laccase activity was assayed using 0.4 mL 10 mM guaiacol in 10% (V/V) acetone containing 1.2 mL 100 mM acetate buffer (pH 5.0) and 0.4 mL enzyme source with appropriate dilution and monitored at 470 nm (*ε* = 6740 M^−1^ cm^−1^) [15]. Lignin peroxidase activity was determined by oxidation of veratryl alcohol in tartrate buffer (pH 2.5) at 310 nm (*ε* = 9,300 M^−1 ^cm^−1^) [16]. MnP activity was assayed using a reaction mixture containing 1 mM guaiacol, 10 mM citrate phosphate buffer (pH 5.5), 1 mM MnSO_4_, and 50 *μ*M H_2_O_2_ at 465 nm [17]. Enzyme activities were expressed in International Units (IU) where one unit corresponded to the amount of enzyme that oxidized one micromole of substrate per minute.

### 2.4. Protein Estimation

An aliquot of culture filtrate of* S. ostrea *with appropriate dilution was used for estimation of soluble protein content according to the Lowry et al. [18]. Bovine serum albumin was used as protein standard.

### 2.5. Statistical Analysis

All the experimental data given in the results were means of triplicates and followed Duncan's new multiple range (DMR) test to find significant difference (*P* ≤ 0.05) between values of each sampling [19].

## 3. Results

All the flasks with growing cultures of* S. ostrea* were withdrawn on alternate days of incubation for measurement of extracellular protein content and enzyme activities in the culture filtrate. Different inducers were screened to get higher enzyme activity from the culture of* S. ostrea*. The inducers included in the present study had diverse effects on enzyme production. Veratryl alcohol at 0.02% exerted maximum inductive effect on the production of three enzymes. Laccase activity is increased by 1.9 times (32,675 U/g of dry substrate) compared to control (17,153 U/g of dry substrate) and an increase of 50% was noted in MnP and LiP production in veratryl alcohol provided flasks. Guaiacol also stimulated the Lcc production by 1.72 times (29,583 U/g of dry substrate) and MnP by 1.4 times (5984 U/g of dry substrate) and has no influence on LiP production. Gallic acid does not have much effect on the production of three enzymes. The remaining three inducers lignin, lignosulfonic acid, and tannic acid had a toxic effect on laccase production. This may be due to the high concentration used in the study. Lignin stimulated LiP by 1.49 times (318.7 U/g of dry substrate) and lignosulfonic acid stimulated MnP by 1.4 times (5,861 U/g of dry substrate) (Table 1).

**Figure 1:**
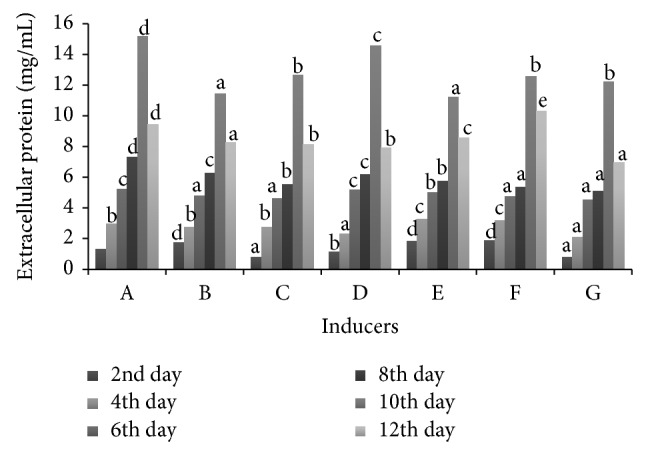
Effect of inducers on secretion of extracellular proteins. A: gallic acid, B: tannic acid, C: guaiacol, D: veratryl alcohol, E: lignin, F: lignosulfonic acid, and G: control. Values are the means of duplicates. Means, in each column, followed by same letter are not significantly different (*P* ≤ 0.05) from each other according to DMR test.

Gallic acid caused maximum secretion of protein by* S. ostrea* followed by veratryl alcohol, guaiacol, and lignosulfonic acid. Low secretion of protein was observed in lignin and tannic acid amended medium compared to control (Figure 1).

**Figure 2:**
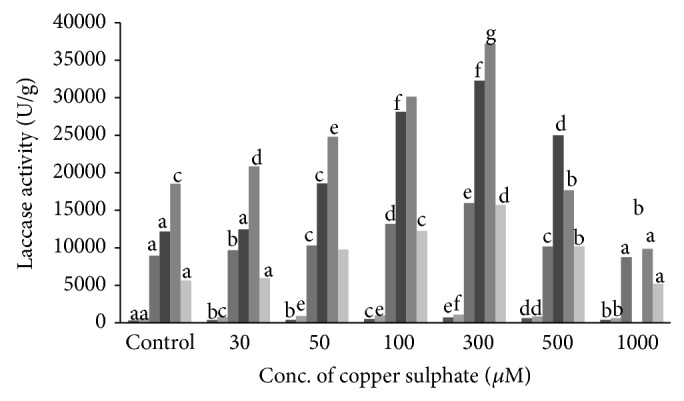
Influence of copper sulphate on laccase production. Values are the means of duplicates. Means, in each column, followed by same letter are not significantly different (*P* ≤ 0.05) from each other according to DMR test.

The effect of copper on Lcc production was determined by growing culture on wheat bran amended with copper sulphate at different concentration, namely, 30, 50, 100, 300, 500, and 1000 *μ*M. The time course of solid state cultures supplemented with different amounts of copper is shown in Figure 2.

**Figure 3:**
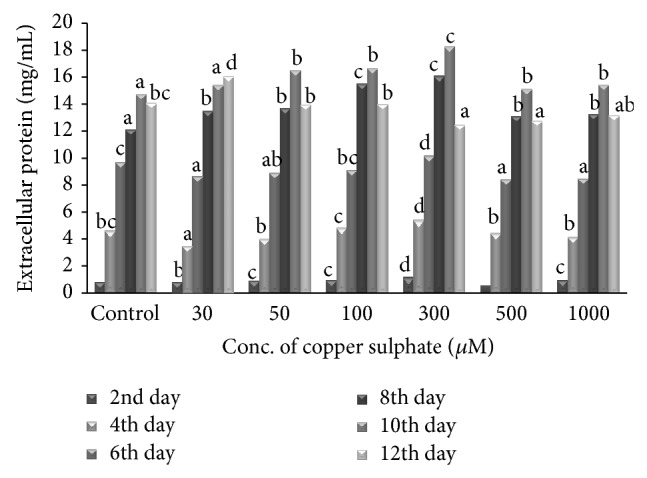
Effect of copper sulphate on secretion of extracellular proteins. Values are the means of duplicates. Means, in each column, followed by same letter are not significantly different (*P* ≤ 0.05) from each other according to DMR test.

The increasing concentration of CuSO_4_ from 30 to 300 *μ*M increased Lcc production. Maximum Lcc activity of 37,182 U/g of dry substrate was obtained on 10th day of incubation. At the same time, control flask (without CuSO_4_) showed Lcc activity of 18,535 U/g of dry substrate which was about 2 times lower than the flask supplemented with 300 *μ*M CuSO_4_. The results clearly show the positive effect of copper sulphate on Lcc production. However when the concentration of copper was increased from 300 to 1000 *μ*M significant decrease in Lcc production was observed. This may be attributed to the inhibitory effect of copper at higher concentrations. Whatever trend observed on Lcc production was also noticed on the influence of CuSO_4_ on secretion of extracellular protein. Maximum extracellular protein of 18.3 mg/mL was released by* S. ostrea* into the medium containing 300 *μ*M CuSO_4_ on 10th day of incubation (Figure 3).

The present study determined the effect of different surfactants on enzyme production. Provision of 1 mL of 1% Tween 80 favored maximum production of ligninolytic enzymes by* S. ostrea*. It exhibited maximum Lcc and MnP activities of 25,109 U/g and 6,303 U/g, respectively, on 10th day of incubation and LiP 252.5 U/g on 12th day of incubation. All the surfactants that are provided in the medium stimulated the production of Lcc and MnP in the order of Tween 80, Tween 20, and Triton X-100. But LiP activity was not enhanced with the addition of Tween 20 and Triton X-100 (Table 2).

**Figure 4:**
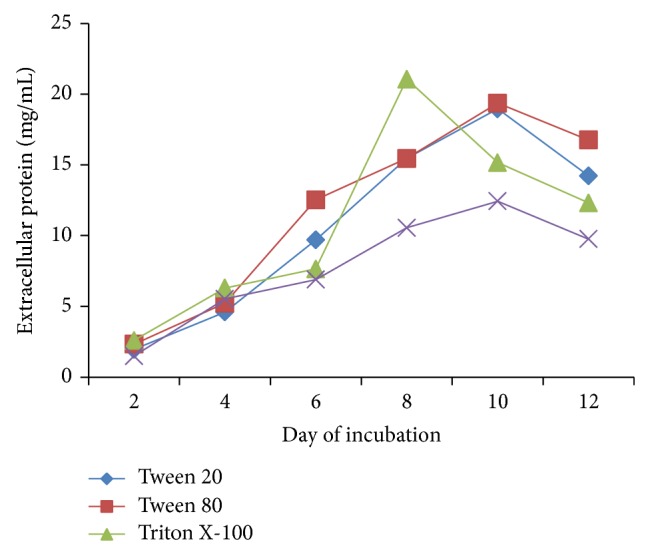
Effect of surfactants on secretion of extracellular protein. Values are the means of duplicates. Means, in each column, followed by same letter are not significantly different (*P* ≤ 0.05) from each other according to DMR test.

Maximum extracellular protein with 21.06 mg/mL was recovered from* S. ostrea* grown on Triton X-100 amended medium on 8th day of incubation followed by Tween 80 (19.36 mg/mL) and Tween 20 (18.96 mg/mL) on 10th day of incubation (Figure 4).

To evaluate optimum volume of Tween 80 required for maximum production of these enzymes different volumes, that is, 0.25, 0.5, 0.75, 1.0, 1.5, and 2.0 mL of Tween 80, were added to the production medium. Enhanced production of Lcc and MnP was observed in the flasks supplemented with 1 mL of Tween 80. 27,055 U/g of Lcc activity and 5,646 U/g of MnP activity were recorded on 10th day of incubation. Maximum production of LiP 302.9 U/g was noted in 1.5 mL Tween 80 supplemented flasks on the same day of incubation Table 3.

Maximum extracellular protein with 18.86 mg/mL was recovered from the 1.5 mL amended medium by* Stereum ostrea* on 10th day of incubation followed by 18.63 mg/mL protein content in the 1 mL Tween 80 supplied medium on the same day of incubation (Table 4).

Dye adsorption abilities of two lignocelluloses, rice bran and wheat bran, were tested by incubating them with two dyes RBB and RBV-5R. The RBB dye adsorbed after 30 min contact time was 80% (40 mg) on wheat bran and 73% (36.5 g) on rice bran whereas 77% (38.5 mg) and 69% (34.5 mg) of RBV-5R were adsorbed onto wheat and rice bran, respectively. The dye adsorbed lignocelluloses were used as growth substrates for enzyme production by* S. ostrea* in solid state fermentation. Both of the dye adsorbed lignocelluloses stimulated the production of laccase. RBV-5R dye adsorbed wheat bran produced the highest laccase of 24,962 U/g on 8th day of incubation compared to control of 13,796 U/g followed by RBV adsorbed rice bran—24,258 U/g (Table 5).

The laccase and MnP activities obtained in RBV-5R adsorbed rice and wheat bran were higher than the activities obtained in RBB adsorbed lignocelluloses. In both the cases not much influence was observed on LiP production. It was reported that addition of inducers and copper induces the laccase production. In the current study, laccase activity values were obtained without any additional mediators or inducers. Among the two substrates tested, RBV-5R adsorbed rice bran was determined to be the best substrate for laccase production and RBV-5R adsorbed wheat bran was found to be the best for MnP production.

**Figure 5:**
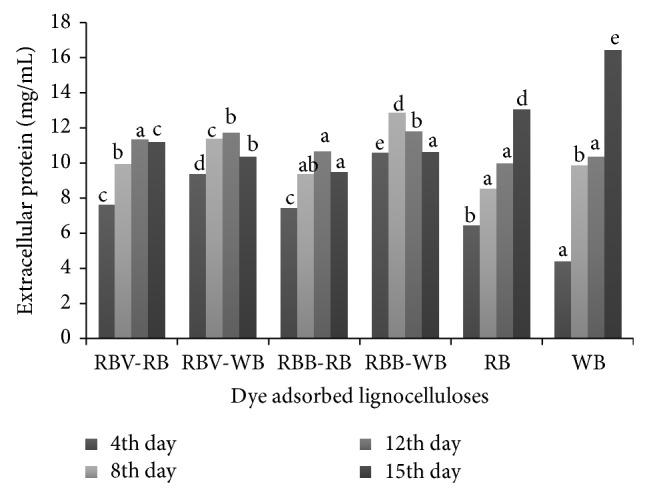
Secretion of extracellular proteins by dye adsorbed lignocelluloses. RBB-RB: remazol brilliant blue adsorbed rice bran, RBB-WB: remazol brilliant blue adsorbed wheat bran, RBV-WB: remazol brilliant violet 5R adsorbed wheat bran, and RBV-RB: remazol brilliant violet 5R adsorbed rice bran. Values are the means of duplicates. Means, in each column, followed by same letter are not significantly different (*P* ≤ 0.05) from each other according to DMR test.

In the initial days of incubation dye adsorbed lignocelluloses secreted high amount of extracellular proteins up to 12 days; later extracellular protein content was found to be decreased. Rice bran and wheat bran alone secreted high amount of protein of 13.05 and 16.41 mg/mL on 15th day of incubation (Figure 5).

## 4. Discussion

SSF has been considered as an efficient method for enzyme production in biotechnological process due to its potential advantages and high yield. In this study we selected ssf using wheat bran, an agro-byproduct containing arabinoxylans and phenolic acids, as a supporting substrate. The white rot fungi,* S. ostrea*, secreted low range of ligninolytic enzymes in submerged fermentation [20] compared to ssf. The ligninolytic activity of white rot fungi depends on many factors, and each strain responds in a particular way to each of these factors. The production of ligninolytic enzymes in ssf was further enhanced by the addition of various inducers, copper sulphate [21], and different surfactants. Phenolic and aromatic compounds such as guaiacol, veratryl alcohol, and ABTS have been widely employed to improve the production of ligninolytic enzymes by several fungal species [22–24]. Results revealed that veratryl alcohol and guaiacol stimulated higher enzyme production compared to other added inducers. The inductive effect of veratryl alcohol and guaiacol on laccase production has been reported by [25]. Guaiacol at 1 mM evidently enhanced the level of laccase production by* Armillariella tabescens *[26]. In this study a stimulating effect of these two compounds on MnP and LiP was observed. With lignosulfonic acid, lignin, and tannic acid, there is a toxicity effect on fungi and process optimization must be performed to consider the utilization of these compounds.

Copper is an essential micronutrient for most living organisms and copper requirements by microorganisms are usually satisfied by low concentrations of metal. However copper present in higher concentration is extremely toxic to microbial cells [27]. In the ascomycete* Podospora anserina*, in which laccase mRNA, amongst others, increased in response to copper and aromatic compounds, it was postulated that laccase acts as a defence mechanism against oxidative stress [28]. This protective function was partly attributed to the chelation of copper ions during synthesis of the laccase enzyme [29]. In* Pleurotus ostreatus *cultures, the presence of copper decreased the activity of an extracellular protease [12]. This might explain the positive effect of copper on enzyme stabilization. Reference [30, 31] reported that the copper at various concentrations stimulates laccase production in* T. pubescens*,* P. eryngii*, and* P. ostreatus*. Our findings are in accordance with those results. Reference [32] observed that laccase activity producing* Phlebia radiata* was increased in media with 1.5 mmol/L of Cu^2+^ while [33] found that optimal concentration of copper ions for laccase production by* Trametes trogii* is 11 mmol/L.

Some studies have demonstrated that the use of surfactants can stimulate fungal growth and enhance enzyme production. Nonionic surfactants such as Tween 80, Tween 20, and Triton X-100 are often considered to be nontoxic and, therefore, do not affect the fungal growth of* S. ostrea*. Several studies of chemical surfactants have shown that charge has an impact on toxicity; cationic surfactants are the most toxic and have been used as antimicrobials. Reference [34] found no negative effect of Tween 80 on* P. chrysosporium* growth. Reference [35] evaluated the toxicity of SDS, Triton X-100, and Tween 80 on fungal strains. The results showed growth inhibition by SDS (anionic surfactant), whereas Triton X-100 and Tween 80 (nonionic surfactants) were well tolerated at the doses evaluated in most of the tested fungi. Several authors have shown an improvement in enzyme excretion in the presence of certain surfactants such as Tween 80 in immobilized and submerged cultures of* P. chrysosporium* [34, 36]. Tween 80 at 0.3 mM greatly enhanced the activities of all the three enzymes by* Ganoderma lucidum* in solid state fermentation of pineapple leaf [37]. Moreover, Tween 80 is known to facilitate the secretion of ligninolytic enzymes [38] and its effect also enhanced LiP production by this selected strain. It was suggested that the surfactants enhance the extracellular enzyme production in filamentous fungi by promoting both the uptake and exit of compounds from the cells through the modification of plasma membrane permeability [34].

Dye adsorption abilities of two types of low cost eco-friendly lignocelluloses, rice bran and wheat bran, were tested. There are studies on biosorption potential of various species of lignocelluloses [39, 40]. Rice bran is a cheap adsorbent for the removal of textile dyes [41]. Kadam et al. [42] used rice bran as a cheap adsorbent for removal of reactive navy blue and reported 90% removal. It was previously reported that dye adsorbed lignocelluloses could be used for the production of ligninolytic enzymes [43]. The result obtained in this study showed that it was possible to use dye adsorbed lignocelluloses for production of ligninolytic enzymes in solid state fermentation. However enzyme production efficiency is strain and substrate dependent [44]. Among the two substrates tested, dye adsorbed wheat bran was determined to be the efficient substrate for the production of ligninolytic enzymes during solid state fermentation. Because wheat bran contains high amount of carbohydrates that can be used as a carbon source, it is a good substrate for fungal growth [45]. It was reported that malachite green adsorbed wheat bran could be used as a solid substrate to produce LiP with* Fomes sclerodermeus* [45].

## Figures and Tables

**Table 1:** Effect of different inducers on ligninolytic enzyme production.

Inducers	Enzymes U/g of dry substrate	Incubation period in days					
		II	IV	VI	VIII	X	XII
Gallic acid (0.02%)	Lcc	492^d^	957^b^	15,655^e^	18,365^f^	6,182^d^	3066^c^
	MnP	126^b^	384^b^	2,164^d^	4,586^f^	2,264^e^	1,062^c^
	LiP	15.3^a^	36.3^b^	49.5^c^	159.0^f^	118.8^e^	52.3^d^

Tannic acid (0.05%)	Lcc	400^b^	858^b^	10,870^e^	12,152^f^	4,432^d^	1,130^c^
	MnP	93^a^	260^b^	1,365^d^	3,563^f^	1,561^e^	462^c^
	LiP	10.8^a^	20.9^b^	143.2^d^	205.4^e^	55.3^c^	21.1^b^

Guaiacol (0.02%)	Lcc	599^e^	1,571^b^	19,895^e^	29,583^f^	9,238^d^	1,828^c^
	MnP	182^d^	343^b^	3,961^e^	5,984^f^	2,982^d^	982^c^
	LiP	11.8^a^	46.2^b^	65.1^c^	189.1^e^	223.9^f^	119.8^d^

Veratryl alcohol (0.02%)	Lcc	562^a^	2,578^b^	21,565^e^	32,675^f^	12,190^d^	1,212^c^
	MnP	133^bc^	390^b^	3,183^e^	6,273^f^	2,164^d^	563^c^
	LiP	12.0^a^	40.5^b^	179.5^e^	309.7^f^	155.8^d^	49.8^c^

Lignin (0.1%)	Lcc	462^c^	1,096^b^	8,401^e^	10,355^f^	3,397^d^	1,158^c^
	MnP	119^ab^	315^b^	1,292^d^	3,180^f^	1,385^e^	655^c^
	LiP	13.2^a^	42.2^c^	152.3^d^	318.7^e^	43.4^c^	39.2^b^

Lignosulfonic acid (0.1%)	Lcc	400^b^	984^b^	8,930^e^	10,186^f^	3,954^d^	1,074^c^
	MnP	109^a^	297^b^	2,256^c^	5,861^e^	2,952^d^	287^b^
	LiP	12.4^a^	31.6^b^	122.8^e^	198.4^f^	69.1^d^	42.9^c^

Control	Lcc	287^a^	1,138^b^	10,152^d^	13,735^e^	17,153^f^	3,941^c^
	MnP	99^a^	287^b^	1,961^c^	2,986^d^	4,161^f^	2,162^e^
	LiP	12.5^a^	37.2^b^	56.7^c^	95.3^d^	211.2^e^	212.8^f^

Values are the means of duplicates.

Means, in each column, followed by same letter are not significantly different (*P* ≤ 0.05) from each other according to DMR test.

**Table 2:** Effect of surfactants on ligninolytic enzyme production.

Surfactant (1%, 1 mL)	Enzymes U/g of dry substrate	Incubation period in days					
		II	IV	VI	VIII	X	XII
Tween 80	Lcc	574^a^	2,285^c^	9,183^c^	17,561^d^	25,109^c^	20,185^d^
	MnP	316^c^	1,231^c^	2,080^bc^	4,084^d^	6,303^c^	3,868^b^
	LiP	34.6^c^	56.3^b^	92.6^c^	113.5^d^	244.9^d^	252.5^d^

Tween 20	Lcc	690^c^	2,773^d^	9,859^d^	14,805^c^	19,667^b^	16,985^b^
	MnP	297^b^	1,505^d^	2,235^c^	3,865^c^	5,692^bc^	5,115^c^
	LiP	77.3^d^	101.5^c^	132.6^d^	180.0^f^	67.9^a^	63.7^b^

Triton X- 100	Lcc	634^b^	1,325^a^	7,694^b^	10,685^a^	20,786^b^	18,993^c^
	MnP	315^c^	621^a^	1,985^a^	3,331^b^	5,115^b^	3,986^b^
	LiP	20.5^b^	35.3^ab^	62.3^b^	92.6^d^	96.4^b^	154.0^c^

Control	Lcc	571^a^	1,524^b^	6,377^a^	12,661^b^	17,189^a^	12,159^a^
	MnP	266^a^	958^b^	1,962^a^	2,834^a^	3,631^a^	3,205^a^
	LiP	10.5^a^	23.5^a^	34.3^a^	126.5^e^	144.7^c^	40.3^a^

Values are the means of duplicates.

Means, in each column, followed by the same letter are not significantly different (*P* ≤ 0.05) from each other according to DMR test.

**Table 3:** Effect of different volumes of Tween 80 on ligninolytic enzyme production.

Volume of 1% Tween 80	Enzymes U/g of dry substrate	Incubation period in days					
		II	IV	VI	VIII	X	XII
0.25 mL	Lcc	300^a^	701^e^	10,275^b^	12,907^b^	20,462^b^	19,338^b^
	MnP	98^a^	273^b^	3,227^ab^	3,936^a^	4,126^a^	3,165^a^
	LiP	28.9^b^	63.4^d^	138.3^d^	162.8^e^	240.8^d^	57.6^c^

0.5 mL	Lcc	301^a^	920^f^	10,653^b^	14,571^c^	23,228^f^	20,636^c^
	MnP	112^ab^	356^c^	3,433^b^	4,160^ab^	5,351^b^	3,563^b^
	LiP	19.5^ab^	50.7^c^	70.0^b^	233.6^e^	155.8^bc^	110.4^d^

0.75 mL	Lcc	361^c^	715^e^	12,834^d^	14,632^c^	26,361^d^	21,162^c^
	MnP	121^b^	274^b^	3,861^bc^	4,360^bc^	5,620^c^	3,628^b^
	LiP	18.4^ab^	40.6^b^	54.8^a^	86.1^b^	141.2^b^	52.0^c^

1 mL	Lcc	350^c^	618^d^	12,261^d^	14,930^c^	27,055^e^	21,362^cd^
	MnP	132^b^	228^ab^	3,636^bc^	4,937^c^	5,646^c^	2,650^a^
	LiP	22.6^b^	39.6^b^	184.7^e^	255.5^f^	162.1^c^	42.4^b^

1.5 mL	Lcc	325^b^	574^c^	13,191^e^	13,335^bc^	25,943^d^	20,652^c^
	MnP	122^b^	209^a^	4,124^c^	4,824^c^	5,602^bc^	3,632^b^
	LiP	11.2^a^	20.5^a^	94.5^c^	105.2^c^	302.9^e^	150.1^e^

2 mL	Lcc	365^c^	524^b^	11,196^c^	12,461^b^	23,532^c^	20,009^bc^
	MnP	105^a^	196^a^	3,915^c^	4,735^b^	5,272^b^	3,133^ab^
	LiP	56.8^c^	130.3^e^	265.4^f^	55.3^a^	26.9^a^	27.5^a^

Control	Lcc	293^a^	496^a^	8,641^a^	10,336^a^	18,334^a^	15,414^a^
	MnP	93^a^	189^a^	2,855^a^	3,583^a^	4,654^ab^	2,935^ab^
	LiP	11.3^a^	27.2^a^	55.4^a^	109.5^c^	125.2^b^	180.6^f^

Values are the means of duplicates.

Means, in each column, followed by the same letter are not significantly different (*P* ≤ 0.05) from each other according to DMR test.

**Table 4:** Effect of different volumes of Tween 80 on secretion of extracellular proteins.

Volume of Tween 80 (mL)	Extracellular protein (mg/mL)					
	II	IV	VI	VIII	X	XII
0.25	1.83^e^	2.86^b^	6.93^b^	12.70^a^	16.60^a^	13.86^b^
0.5	1.62^d^	2.90^b^	6.96^b^	13.93^b^	17.20^ab^	14.10^bc^
0.75	0.86^a^	3.64^c^	7.13^c^	14.00^b^	18.50^c^	13.42^b^
1.0	0.94^a^	3.6^c^	6.9^b^	14.52^c^	18.63^c^	12.56^a^
1.5	1.10^b^	3.73^c^	7.50^d^	13.20^ab^	18.86^d^	15.26^d^
2	1.40^c^	2.60^a^	6.66^a^	12.60^a^	16.26^a^	12.80^a^

Values are the means of duplicates.

Means, in each column, followed by the same letter are not significantly different (*P* ≤ 0.05) from each other according to DMR test.

**Table 5:** Effect of dye adsorbed lignocelluloses on ligninolytic enzyme production.

Lignocelluloses	Enzymes U/g of dry substrate	Incubation period in days			
		IV	VIII	XII	XV
RBV-RB	Laccase	1669^c^	18,653^d^	24,258^f^	11,765^d^
	MnP	819^d^	2952^d^	3,653^c^	1,345^bc^
	LiP	23.2^a^	86.5^b^	120.6^a^	118^b^

RBV-WB	Laccase	1,906^d^	24,962^f^	19,545^e^	13,531^e^
	MnP	1,104^e^	3,895^e^	3,793^c^	1,843^d^
	LiP	25.8^a^	72.8^a^	143.9^c^	270.9^e^

RBB-RB	Laccase	573^a^	7,253^b^	10,162^b^	6,238^b^
	MnP	325^b^	1,362^a^	1,956^a^	1,063^b^
	LiP	N^D^	75.3^a^	110.6^a^	118.8^b^

RBB-WB	Laccase	2,237^e^	23,096^e^	17,368^d^	10,632^c^
	MnP	1,235^e^	4,652^f^	3,056^b^	1562^c^
	LiP	128.1^b^	180.6^d^	126.4^ab^	36.5^a^

Rice bran	Laccase	653^ab^	6,036^a^	8,635^a^	5,185^a^
	MnP	125^a^	1,814^b^	2,092^a^	876^a^
	LiP	12.8^a^	85.4^b^	115.5^a^	160.0^d^

Wheat bran	Laccase	1,263^b^	12,550^c^	13,796^c^	10,534^c^
	MnP	552^c^	2,272^c^	2,982^b^	1,292^b^
	LiP	21.6^a^	112.6^c^	120.3^a^	144.4^c^

RBB-RB: remazol brilliant blue adsorbed rice bran, RBB-WB: remazol brilliant blue adsorbed wheat bran, RBV-WB: remazol brilliant violet 5R adsorbed wheat bran, and RBV-RB: remazol brilliant violet 5R adsorbed rice bran.

Values are the means of duplicates.

Means, in each column, followed by the same letter are not significantly different (*P* ≤ 0.05) from each other according to DMR test.
